# Bibliographical Notices

**Published:** 1851-04

**Authors:** 


*The Anatomy, Physiology and Pathology of the Eye.* By Henry Howard, M. R. C. L. S., Surgeon to the Montreal Eye and Ear Istitution.

In the midst of the abundance of medical literature, with which the press is teeming, it is truly refreshing to meet with a work, which comes honestly up to its avowed purpose, in which the author turns neither to the right nor to the left to make popular capital, and which, though evidently the result of much study and observation, is uncontaminated by the slightest tinge of parade or pedantry.

Dr. Henry Howard, as Surgeon to the Eye and Ear Institution, at Montreal, has enjoyed the advantage of a wide field for practice, an advantage of which he seems to be in every way worthy, for, as a result, he has given us an excellent book. We regret that want of time and space prevents our entering into its minutiæ, but assure our readers, that a perusal will amply repay their trouble.

The book commences with the anatomy of the eye, bones, muscles, nerves, membranes, &c., with clear and concise descriptions, correct, and withal sufficiently minute for every purpose. The physiology of the nerves, muscles, &c., follows, in which science the author appears to be fully up with the best physiologists of the day. He differs with some of them on a few minute points, and supports his opinions by facts taken from close observation, with considerable skill and judgment, but as time alone can settle many of these points, we shall offer no opinion on them.

The pathology of the eye comes next; the causes, symptoms and treatment of the different morbid conditions of the organ are described with great accuracy and clearness. The chapter on simple, severe and purulent ophthalmia, we call attention to, as being particularly worthy of careful perusal. The section on periosteal disease of the orbit, is good, and there are some interesting remarks on inflammation of the tunica vaginalis oculi.

In the course of the volume, almost all the approved operations on the eye are faithfully described, but the organ has been sliced and cut in such variety of modes, that originality of carving is next to impossible.

An instrument invented by the author, for making the section of the cornea in the operation of extraction is described; he says that it possesses the great advantage of dividing the cornea without endangering the iris, in case of its falling forwards; it likewise obviates the necessity of making a counter-puncture, which all surgeons admit to be the most difficult part of the operation. Those interested in the subject, will do well to read and judge for themselves; our limits will not permit us to make explanatory quotations, nor, to speak candidly, do we place much reliance on such toys, for we have always thought with Liston, that a quick eye, steady hand, and a sharp knife, are more to be relied on than all the grooves, slides, springs, and guillotines that have ever been invented.

The book is well gotten up, and handsomely printed. We advise all who are interested in the subjects of which it treats to procure it; the practitioner may often refer to it with advantage, and the student cannot be better employed than in reading it attentively.

---

*On the Use and Abuse of Alcoholic Liquors, in Health and Disease. Prize Essay.* By William B. Carpenter, M. D., F. R. S., F. G. S., &c. &c. Philadelphia, Lea & Blanchard. pp. 204.

The important nature of the subject discussed in this Essay, and the distinguished reputation of its author, are of themselves sufficient to secure for it a large share of attention, both in and out of the medical profession. Dr. Carpenter approaches the subject as a man of science, and not as an advocate anxious to establish preconceived views. That he has fully succeeded in his effort to support this character, we are not prepared to affirm, but if his bias as a moralist has induced him to accept testimony which rigid science would reject, a careful perusal of this essay has convinced us that it is worthy an examination by the best informed on the subjects it relates to.

In the preface to the work, the reader is requested "to keep the following issues in view, as those to which he [the author,] is desirous of leading him."

"In the *first* place. That, from a scientific examination of the *modus operandi* of Alcohol upon the Human body, when taken in a *poisonous* dose, or to such an extent as to produce Intoxication, we may fairly draw inferences with regard to the specific effects which it is likely to produce, when repeatedly taken in excess, but not immediately to a fatal amount."

"*Secondly*. That the consequences of the *excessive* use of Alcoholic liquors, as proved by the experience of the Medical Profession, and universally admitted by medical writers, being precisely such as the study of its effects in poisonous and immediately fatal doses would lead us to anticipate, we are further justified in expecting that the habitual use of smaller quantities of these liquors, if sufficiently prolonged, will ultimately be attended, in a large proportion of cases, with consequences prejudicial to the human system,—the morbid actions thus engendered being likely rather to be chronic, than acute, in their character.

"*Thirdly*. That as such morbid actions are actually found to be among the most common disorders of persons advanced in life, who have been in the habit of taking a 'moderate' allowance of alcoholic liquors, there is very strong ground for regarding them as in great degree dependant upon the asserted cause; although the long postponement of their effects may render it impossible to *demonstrate* the existence of such a connection.

"*Fourthly*. That the preceding conclusion is fully borne out by the proved results of the 'moderate' use of Alcoholic liquors, in producing a marked liability to the acute forms of similar diseases in hot climates, where their action is accelerated by other conditions; and also by the analogous facts now universally admitted, in regard to the remotely injurious effects of slight excess in diet, imperfect aeration of the blood, insufficient repose, and other like violations of the Laws of Health, when habitually practiced through a long period of time.

"*Fifthly*. That the capacity of the healthy Human system to sustain as much bodily or mental labor as it can be legitimately called upon to perform, and its power of resisting the extremes of Heat and Cold, as well as other depressing agencies, are not augmented by the use of Alcoholic liquors; but that, on the other hand, their use, under such circumstances, tends positively to the impairment of that capacity.

"*Sixthly*. That, where there is a deficiency of power, on the part of the system, to carry on its normal actions with the energy and regularity which constitute health, such power can rarely be imparted by the habitual use of Alcoholic liquors; its deficiency being generally consequent upon some habitual departure from the laws of health, for which the use of Alcoholic liquors cannot compensate; and the employment of such liquors, although with the temporary effect of palliating the disorder, having not merely a remotely injurious effect *per se*, but also tending to mask the action of other morbific causes by rendering the system more tolerant of them.

"*Seventhly*. That, consequently, it is the duty of the Medical Practitioner to discourage as much as possible the *habitual* use of Alcoholic liquors, in however 'moderate' a quantity, by all persons in ordinary health; and to seek to remedy those slight departures from health which result from the 'wear and tear' of active life, by the means which shall most directly remove or antagonize their causes, instead of by such as simply palliate their effects.

"*Eighthly.* That, whilst the habitual use of Alcoholic liquors, even in the most 'moderate' amount, is likely (except in a few rare instances) to be rather injurious than beneficial, great benefit may be derived, in the treatment of Disease, from the *medicinal* use of Alcohol in appropriate cases; but that the same care should be employed in the discriminating selection of those cases, as would be taken by the conscientious practitioner in regard to the administration of any other powerful remedy which is poisonous in large doses."

If the manner in which Dr. Carpenter argues these "issues" does not amount to an actual demonstration of their truth, he will, we think, cause the conscientious practitioner to hesitate before, by his advice or otherwise, he encourages a violation of the principles laid down in them. Independently of the question of morality which the subject involves, Dr. Carpenter's work possesses much interest for the Phrenologist, the Pathologist, and the general reader.

Amongst the various positions considered by our author, that which refers to the "general effect of the excessive use of alcoholic liquors on the duration of life," is an important one to the medical practitioner—and especially to those connected with life assurance institutions. Dr. C. says:

"At the age of 40 years, the annual rate of mortality among the whole population of England, is about 13 per 1000: whilst among the lives insured in Life Offices, it is about 11 per 1000; and in those in Friendly Societies, is about 10 per 1000. Now the average mortality of *all ages* between 15 and 70 years, is about 20 per 1000; whereas in the Temperance Provident Institution, after an experience of eight years, and with several lives above 70 years of age, the average mortality has has been only 6 per 1000, up to the present season, in which it has undergone a slight increase from the Cholera epidemic."

The division which refers to the "*supposed use of alcohol in sustaining the vital powers*" is especially interesting, the author having drawn together a copious collection of illustrations from actual experience to prove his positions.

He considers this question under the heads; 1st, Of severe *bodily exertion*, 2d, of severe *mental* exertion; 3d, of extreme *cold*; 4th, of extreme *heat*; 5th, of *morbific agencies.*

And under all these heads he gives weighty reasons for believing that alcohol in any quantity, as a rule, exerts pernicious, rather than beneficial influences. Numerous examples under a variety of circumstances, are brought to prove, that when moderate spirit drinkers and total abstinence men are brought together under exposure or severe bodily labor, the results are uniformly in favor of the latter.

Under the head of *severe mental exertion*, after admitting that a particular kind of genius may occasionally be excited to brilliancy by the stimulus of alcohol, Dr. C. gives the following result of his own experience:

"We do not usually find that the men most distinguished for that combination of intellectual powers which is known as *talent*, are disposed to make such use of Alcoholic stimulants for the purpose of augmenting their mental powers; for that spontaneous activity of the mind itself, which it is the tendency of alcohol to excite, is not favorable to the exercise of the observing and purely reasoning faculties, or to the steady devotement of concentrated attention to any subject which it is desired to investigate profoundly. Of this we have a remarkable illustration in the habits of practiced gamblers; who, when about to engage in contests requiring the keenest observation and the most sagacious calculations, and involving an important stake, always 'keep themselves cool,' either by entire abstinence from fermented liquors, or by the use of those of the weakest kind in very small quantities. And we find that the greatest part of that intellectual labor which has most extended the domain of human knowledge, has been performed by men of remarkable sobriety of habit, many of them having been constant water-drinkers. Under this last category, it is said, may be ranked Demosthenes and Haller; Dr. Johnston, in the latter part of his life, took nothing stronger than tea, while Voltaire and Fontenelle used coffee; and Newton and Hobbes were accustomed to solace, not to excite, themselves by the fumes of tobacco. In regard to LOCKE, whose long life was devoted to constant intellectual labor, and who appears, independently of his eminence in his special objects of pursuit, to have been one of the best informed men of his time, the following very explicit and remarkable testimony is borne by one who knew him well. 'His diet was the same as other people's, except that he usually drank nothing but water; and he thought that his abstinence in this respect had preserved his life so long, although his constitution was so weak.'

"Having for several years past, been himself performing an amount of steady mental labor, which to most persons would appear excessive, the writer may be allowed to refer to his own experience, which is altogether in favor of Total Abstinence from alcoholic liquors, as a means of sustaining the power of performing it. Having been brought up as a water-drinker, he never accustomed himself to the habitual use of alcoholic liquors, scarcely ever tasting them, except when occasionally led to do so by social influences, or when he believed that a small amount of stimuus would improve the 'tone' of his system, which is liable to a peculiar relaxation in certain states of the atmosphere. On determining, about four years since, to give up the occasional use of wine, &c., as a social indulgence, he still held himself free to employ it when he might think it likely to increase the general powers of his system; and for some time he continued to have occasional recourse to alcoholic stimulants (never exceeding a single glass of wine, or half a tumbler of bitter ale,) when he felt himself suffering under the peculiar depression just referred to. He gradually, however, found reason to doubt the utility of the remedy, and has for the last two years entirely given it up. During these two years, he has gone through a larger amount of mental labor than he ever did before in the same period of time; and he does not hesitate to say that he has performed it with more ease to himself than on his former system; and that he has been more free than ever from those states of depression of mental energy which he was accustomed to regard as indicating the need of a temporary support to antagonize the depressing cause. In fact, he now finds that, when these do occur, the use of alcoholic stimulants (taken even in small amount) is decidedly injurious to him; diminishing rather than increasing his power of mental exertion at the time, and leaving him still less disposed for it after their effect has gone off. He attributes this change to his entire disuse of alcoholic liquors under all other circumstances; and he cannot but believe that the results which he now experiences, and which have led him to relinquish these stimulants altogether, are the *natural* effect of them upon the healthy system; and that the benefit which some persons consider themselves as deriving from their use, arises from their simply removing *for a time* the depression which results (at a long interval, it may be) from their *previous* employment."

In conclusion, we will again repeat our anxiety to bring this little volume under the notice of the profession.

---

*Ether and Chloroform, their employment in Surgery, Dentistry, Midwifery, Therapeutics, &c.* By J. F. B. Flagg, M. D., Surgeon Dentist, &c. Philadelphia, Lindsay & Blakiston, 1851.

The little work of Dr. Flagg presents an excellent resumé of the history of ether and chloroform, together with their physiological, psychcological and therapeutical effects. We commend it to all who may desire information on these various points, or as to the best and safest modes of administering it. It comes from one who has had great and extended experience in its use.

